# Facelift Under Local Anesthesia: A Retrospective Single-Center Series of 117 Patients Undergoing Deep Plane, High SMAS, and Coronal Rhytidectomy

**DOI:** 10.1093/asjof/ojag166

**Published:** 2026-09-10

**Authors:** Mariagiovanna Lombardi, Martina Ponzo, Eric Santamaria, Fibian Chaterinee Luna Aguilar, Francesco D’Andrea, Humberto Gustavo Martìnez, Luis Raùl Cajamarca Bermeo, Jesus Carlos Ceballos Villalva, Nicolàs Ricardo Brito, Pier Paolo Perrella, Ludovica De Gregorio, Daniela Guerrero Salazar, Lida Mendoza Andrade, Enrique Chávez Serna

## Abstract

**Background:**

Facelift surgery is increasingly performed in an outpatient setting, and local anesthesia with minimal sedation may reduce the anesthesiologic burden while preserving patient cooperation. However, the safety of local-anesthesia pathways for procedures of varying extent, including deep plane rhytidectomy, high SMAS facelift, and coronal rhytidectomy, remains incompletely characterized.

**Objectives:**

To evaluate the feasibility and early safety of a standardized local-anesthesia pathway in a single-center retrospective series of primary aesthetic cervicofacial lifting procedures and to contextualize the findings within the existing literature on awake facial rejuvenation.

**Methods:**

A retrospective analysis was conducted of consecutive primary aesthetic facelifts performed under local anesthesia between March 6, 2024, and January 14, 2026.

**Results:**

Overall, 117 procedures were included: 90 deep plane facelifts, 6 high superficial musculoaponeurotic system (SMAS) facelifts, and 21 coronal rhytidectomies. The cohort included 110 women (94.0%) and 7 men (6.0%), with a mean age of 56.53 ± 7.92 years and a mean BMI of 24.6 ± 3.1 kg/m^2^. Concomitant procedures were performed in 16 patients (13.7%). The mean operative time was 5.0 ± 0.6 hours (range, 3.5-8.0 hours). No case required conversion to general anesthesia or early termination. Six complications were documented (6/117, 5.13%), all occurring in the deep plane subgroup (6/90, 6.7%).

**Conclusions:**

In selected patients, primary facelift surgery under local anesthesia appears feasible across a broad spectrum of cervicofacial lifting techniques when performed within a structured pathway including preoperative screening, standardized infiltration, maintenance of verbal responsiveness, and careful hemodynamic monitoring. Patient selection, procedural predictability, and proactive control of anxiety, pain, blood pressure, and nausea/vomiting are likely more important than any single anesthetic formula.

**Level of Evidence: 4 (Therapeutic):**

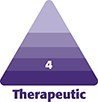

Cervicofacial facelift remains a cornerstone procedure in surgical facial rejuvenation.^1^ Modern facelift surgery encompasses a spectrum of techniques, including skin-SMAS approaches, high SMAS rhytidectomy, deep plane dissection,^2^ and coronal or temporal lifting procedures, each characterized by different dissection patterns, operative duration, and perioperative stimulation. With the evolution of technical approaches, anesthetic strategy has become an important determinant of patient experience, outpatient logistics, hemodynamic control, and complication risk. General anesthesia provides airway control and immobility but may be associated with longer recovery, postoperative nausea and vomiting, greater resource utilization, and a physiological stress profile that may not be necessary for every selected aesthetic patient. Conversely, local anesthesia, with or without minimal sedation, may facilitate outpatient surgery, preserve patient responsiveness, and potentially improve hemodynamic stability when anxiety and pain are adequately controlled. The main question is whether patient comfort and procedural control can be maintained during longer or more invasive procedures without compromising safety. The central safety endpoint in facelift surgery is hematoma. Its occurrence is influenced not only by surgical hemostasis but also by perioperative hypertension, pain, anxiety, coughing, nausea, vomiting, and medication-related bleeding risk.^3,4^ Protocols vary widely, ranging from isolated local infiltration to combinations of oral benzodiazepines, nerve blocks, topical anesthesia, diluted lidocaine with epinephrine, tumescent solutions, and intravenous sedation.^5-10^ The literature on awake or local-anesthesia facelift surgery is heterogeneous: some studies focus on limited lower-face or temporal procedures, whereas others describe broader office-based or deep plane experiences.^11-16^

In this context, the present study aims to evaluate feasibility and early safety in 117 consecutive primary aesthetic rhytidectomies performed using a standardized local-anesthesia pathway, including deep plane, high SMAS, and coronal procedures.

## METHODS

### Single-Center Case Series

A retrospective single-center analysis was conducted of aesthetic facelift procedures performed under local anesthesia between March 6, 2024 and January 14, 2026. All consecutive cases available during the study period were included. Only primary facelift cases were performed at our institution during the study period; no secondary/revision facelifts were included. The variables collected included procedure type (deep plane, high SMAS, and coronal), demographic characteristics (sex and age), presence of concomitant procedures during the same operative session and documented complications. The presence of associated procedures was defined as “multiprocedure” when the operative description reported at least 2 procedures identified by the presence of the separator “+”. During the preoperative pathway, all patients underwent history taking and physical examination with verification of comorbidities, allergies, particularly to local anesthetics, current medications, and supplements. Patients were instructed to discontinue for 2 weeks any substances associated with an increased risk of bleeding or hematoma and to avoid aspirin and nonsteroidal anti-inflammatory drugs for at least 7 days, while continuing chronic antihypertensive and antidiabetic therapies as prescribed including on the day of surgery. Patients were asked to arrive at least 60 minutes before the procedure and compliance with premedication was verified: prophylactic antibiotic therapy with amoxicillin/clavulanic acid 875/125 mg, etamsylate 500 mg on the evening before surgery and on the morning of surgery, pregabalin 75 mg on the morning of surgery and paracetamol 1 g on the evening before surgery and on the morning of surgery.

Operative data extraction and procedure classification were performed by a single investigator using the official operative report for each case. The “+” separator reflects the standardized way procedures are listed in the operative description; when any ambiguity was present, the full operative note was reviewed to confirm the exact procedures performed and to ensure complete capture of concomitant procedures. In our single-center cohort, procedures included deep plane, high SMAS and coronal cervicofacial lifting (all primary cases). Concomitant minor procedures (eg, blepharoplasty, lip lift) were recorded when performed during the same operative session.

Complications were defined as adverse events documented within the first 15 postoperative days. Hematoma was categorized according to clinical relevance when details were available: minor hematoma/bleeding managed conservatively, hematoma requiring evacuation or drainage in the outpatient setting, and hematoma requiring operative exploration.^3,4,11^ Other complications were recorded by event type, timing, treatment, and outcome.

Intraoperative monitoring included continuous pulse oximetry and heart rate monitoring, as well as intermittent blood pressure measurements using an automatic cuff. The patient was positioned supine, and verbal responsiveness was maintained throughout the procedure in order to monitor comfort, cooperation, level of consciousness, and airway patency.

Oral benzodiazepine premedication consisted of 0.5 mg of alprazolam administered orally 40 minutes before surgery. After antisepsis and sterile draping infiltration was performed using a premixed solution composed of 250 mL of 0.9% normal saline, 50 mL of 2% lidocaine, 20 mL of ropivacaine 7.5 mg/mL, and 1 ampoule of epinephrine 1 mg/mL. Infiltration was carried out with a 27G needle according to a standardized sequence: first a portion was injected in the cervical area, and this was followed by facial and cervical infiltration on each side alternating sides in order to take advantage of the onset time while closing the first treated side. Discomfort was managed with additional oral analgesics or targeted infiltration; verbal stimulation was maintained throughout the procedure to assess the level of consciousness and airway patency.^9,10^

The neck was opened/dissected when indicated by the planned cervicofacial procedure, including cases requiring cervical work through a submental incision and platysma plication (67/90) or elevation of a cervicofacial flap through an exclusively lateral cervical approach (23/90). No patient underwent neck lipofilling. Postoperative follow-up was scheduled on postoperative Day 1, Day 3, at 1 week, 2 weeks, 1 month, 4 months, and 1 year. Follow-up assessments were performed in the outpatient clinic by physical examination. For the purposes of this study, complications were defined and recorded within the first 15 postoperative days. All patients completed at least 1 year of follow-up in the available dataset.

### Ethics Statement

This retrospective analysis was conducted in accordance with the Declaration of Helsinki. Written informed consent for surgery was obtained from all patients; separate consent for the use of clinical photographs in scientific publication was obtained for the representative images presented.

## RESULTS

### Results of the Single-Center Case Series

During the observation period, a total of 117 procedures were included, consisting of 90 deep plane (Figure 1), 6 high SMAS and 21 coronal procedures. The sample included 110 women (94.0%) and 7 men (6.0%). Mean age was 56.53 ± 7.92 years with a range of 30-76 and age data were available for 114 of 117 cases. Mean BMI was 24.6 ± 3.1 kg/m^2^, with most patients falling within the normal-weight or mildly overweight range. Overall, 21 patients (17.9%) were active smokers and 14 patients (12.0%) were former smokers. The most frequently recorded comorbidities were arterial hypertension in 18 patients (15.4%), thyroid disease in 11 patients (9.4%), dyslipidemia in 10 patients (8.5%), and type 2 diabetes mellitus in 4 patients (3.4%). Most patients were classified as ASA I or ASA II, while no patient with uncontrolled systemic disease or major anesthetic contraindications was included in the local-anesthesia pathway (Table 1). Concomitant procedures performed during the same operative session (“multiprocedure”) were observed in 16 of 117 cases (13.7%). The frequency of multiprocedure cases was higher in the coronal subgroup (8/21; 38.1%) than in the deep plane subgroup (7/90; 7.8%) and the high SMAS subgroup (1/6; 16.7%). The most frequent combination was “coronal rhytidectomy + upper and lower blepharoplasty + lip lift” (*n* = 3); all other combinations were sporadic (*n* = 1 each). In 86 patients undergoing deep plane facelift, 12-Fr closed-suction drains were placed through the end of the posterior auricular incision, in a subcutaneous plane, and advanced to just short of the cervical midline. In the remaining 4 patients, a 3-0 Prolene suture alone was used to create a hemostatic net, positioned inferior to the mandibular border; additional sutures were added as needed in a vertical pattern over the face. A light compressive dressing was then applied, and the patient was discharged home. Drains and the hemostatic net were generally removed after a mean of 2 days. Because this study evaluates feasibility and early safety of a standardized local-anesthesia pathway in real-world aesthetic facial rejuvenation, procedures were not restricted to a single lifting technique; internal validity is therefore interpreted at the level of the anesthesia pathway rather than technique-specific comparative effectiveness. Mean operative duration was 5.0 ± 0.6 hours (range 3.5-8.0 hours). All cases were completed under local anesthesia without conversion to general anesthesia or early termination. All patients completed follow-up through at least 1 year (0% loss to follow-up). Complications were defined as adverse events first documented within the first 15 postoperative days; subsequent clinical evolution and resolution were recorded during scheduled follow-up. Overall, 6 complications were documented (6/117, 5.13%) (Table 2). All occurred in the deep plane subgroup. No complications were recorded in the high SMAS or coronal subgroups. Specifically, 1 postoperative hematoma occurred and was surgically drained the following day, without clinical or aesthetic sequelae; 3 small retroauricular skin necroses measuring ≤0.5 cm were managed conservatively with local wound care and healed by secondary intention without surgical revision; 1 small granuloma was treated in the outpatient setting with local care and removal of the offending suture material, with complete resolution; and 1 transient, mild traction-related weakness in the zygomatic branch territory, consistent with neuropraxia, was observed. The latter was managed with clinical monitoring and showed complete spontaneous recovery within 3 months. No early surgical-site infections or major adverse events were observed.

**Figure 1.**
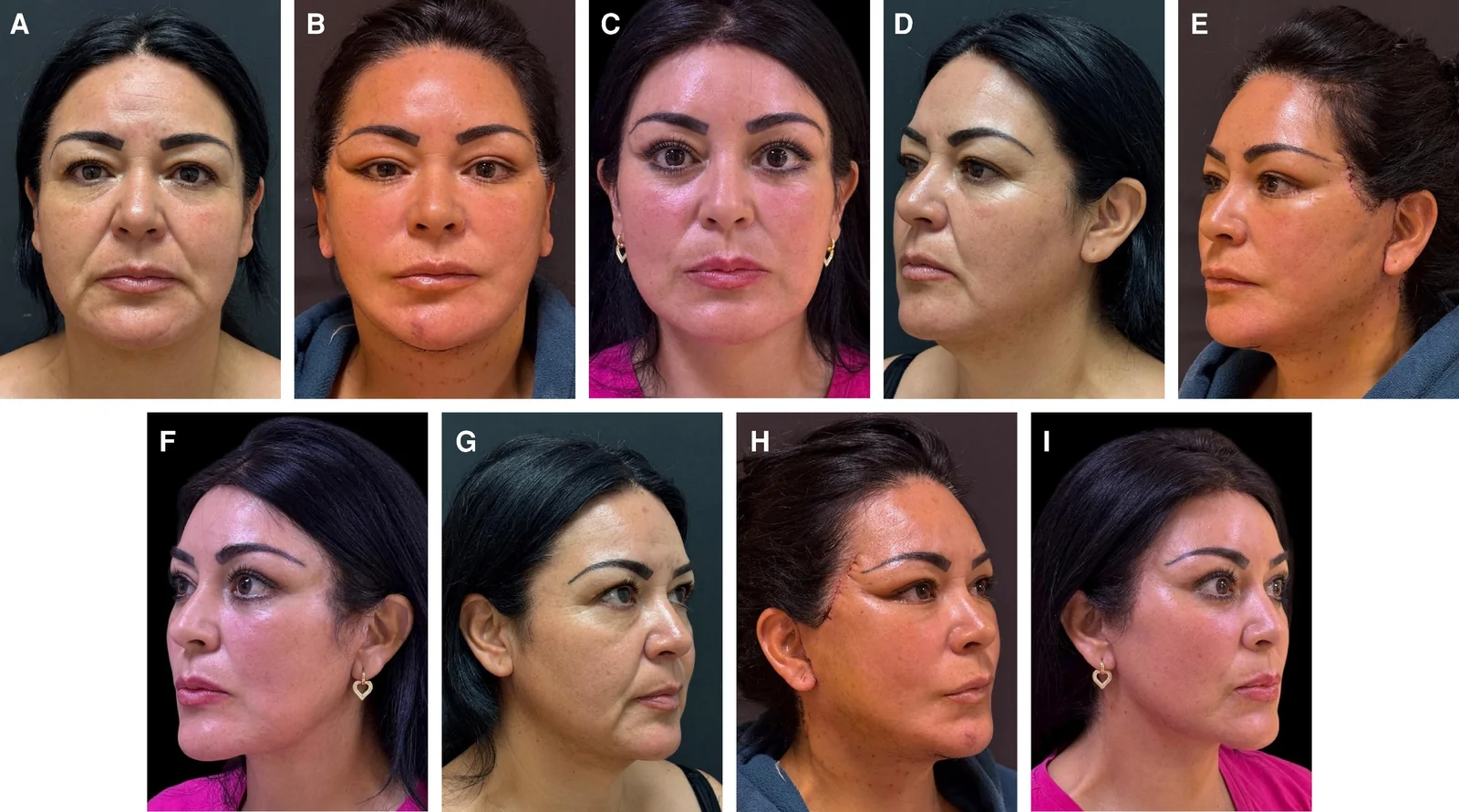
Representative result of deep plane facelift performed under local anesthesia in a 52-year-old female patient. (A, D, G) Preoperative photographs, (B, E, H) postoperative Day 1 photographs, and (C, F, I) 1-year follow-up photographs are shown. (A) Preoperative frontal view. (B) Postoperative Day 1 frontal view. (C) One-year postoperative frontal view. (D) Preoperative left lateral/oblique view. (E) Postoperative Day 1 left lateral/oblique view. (F) One-year postoperative left lateral/oblique view. (G) Preoperative right oblique view. (H) Postoperative Day 1 right oblique view. (I) One-year postoperative right oblique view. Standardized views demonstrate early postoperative changes with preserved facial contour and, at 1-year follow-up, improved jawline definition, cervicomental angle, and midface-neck harmony, with well-concealed scars and a natural overall rejuvenation.

**Table 1.** Demographic Characteristics of the Study Cohort

Characteristics	Value
Patients/procedures, *n*	117
Female sex, *n* (%)	110 (94.0%)
Male sex, *n* (%)	7 (6.0%)
Age, mean ± SD (range), years	56.53 ± 7.92
BMI, mean ± SD, kg/m^2^	24.6 ± 3.1
Active smokers, *n* (%)	21 (17.9%)
Former smokers, *n* (%)	14 (12.0%)
Arterial hypertension, *n* (%)	18 (15.4%)
Thyroid disease, *n* (%)	11 (9.4%)
Dyslipidemia, *n* (%)	10 (8.5%)
Type 2 diabetes mellitus, *n* (%)	4 (3.4%)
ASA I, *n* (%)	65 (55.6%)
ASA II, *n* (%)	52 (44.4%)
ASA III or higher, *n* (%)	0 (0%)

**Table 2.** Specific Complications Recorded in the Cohort

Complication	*n*	% of total cohort	Subgroup	Management	Outcome
Postoperative hematoma	1	0.85%	Deep plane	Surgical drainage on postoperative Day 1	No clinical or aesthetic sequelae
Retroauricular skin necrosis ≤0.5 cm	3	2.56%	Deep plane	Conservative treatment with local wound care	Healed by secondary intention; no surgical revision required
Small granuloma	1	0.85%	Deep plane	Outpatient local care/removal of the offending suture material	Complete resolution
Transient mild weakness in the zygomatic branch territory, consistent with neuropraxia	1	0.85%	Deep plane	Clinical monitoring and supportive medical therapy	Complete spontaneous recovery within 3 months
Early surgical-site infection	0	0%	—	—	Not observed
Major adverse events	0	0%	—	—	Not observed
Total complications	6	5.13%	All deep plane	—	—

## DISCUSSION

This review shows that “facelift under local anesthesia” is not a single technique but rather a group of strategies that share a common goal: to perform lifting procedures while keeping the patient cooperative, with acceptable comfort and with control of the hemodynamic determinants that influence bleeding complications.^3-5,8^ When contextualizing the findings of our single-center series within the available literature, it becomes clear that direct comparison across studies remains complex. This difficulty is mainly related to the heterogeneity of the surgical procedures included, the different levels of sedation used, and the lack of uniform outcome definitions. These factors may contribute to the variability in hematoma rates reported in the literature and should be considered when interpreting our results.^4,11,15,16^

With regard to patient selection, the literature consistently indicates that the success of local anesthesia depends less on the anesthetic agent itself than on the match between patient profile and procedural complexity.^5,8^ Tolerance of an awake procedure, psychological cooperation, and predictability of operative time repeatedly emerge as central determinants of feasibility.^5,11,16^

From a practical standpoint, awake facial rejuvenation is best suited to patients who are able to remain cooperative, with manageable anxiety and a predictable operative burden. General anesthesia should be favored when cooperation is unlikely, for example in cases of severe anxiety or panic; when hemodynamics are difficult to control, for example in poorly controlled hypertension; when airway management concerns predominate; or when the expected extent or duration is high or unpredictable, particularly in complex multiprocedure or revision cases.

Focused series involving temporal or midface lifting, cheek lift, and minilifting of the lower third of the face and anterior cervical neck indirectly support this concept, as they generally report local or transient complications in settings where surgical extent is limited.^12-14^ More recent evidence suggests, however, that this boundary may be broader than previously assumed, since awake surgery has also been reported for deep plane facelift with extended deep neck contouring when perioperative management is highly standardized and the procedure is performed by experienced surgeons.^15^

This reinforces the idea that the practical threshold for local anesthesia is not defined by the procedure name alone, but by the combined burden of dissection, neck work, duration, and patient tolerance. In this context, the authors’ single-center series adds a pragmatic element: despite including a high proportion of deep plane procedures, the cohort maintained a demographic profile similar to that of other aesthetic series, with female predominance and a mean age around 50-60 years, while still including a non-negligible proportion of combined procedures. This suggests that local anesthesia may also be applied in real-world pathways of variable complexity when patient selection and preoperative preparation are structured.

As for the anesthetic protocol, the literature suggests several nonmutually exclusive approaches: standardizing infiltration, broadening sensory coverage through regional blocks, improving comfort through topical anesthesia or oral premedication, and using diluted high-volume or tumescent infiltration to facilitate both analgesia and dissection.^5-7,14,15^

The advantage of protocol standardization lies in reproducibility, simplification of dosing logic, and reduction of technical variability, provided that cumulative dose limits and physiologic monitoring are respected.^5,8^ Block-based strategies may reduce infiltration needs, whereas infiltration-centered strategies may simplify workflow and maintain a more uniform operative sequence.^6,7^ Tumescent variants further widen the spectrum by showing that the same anesthetic philosophy can be applied not only to limited lower-face and neck procedures but also to more complex deep plane and cervical dissections in expert hands.^14,15^ Across all models, sedation remains a continuum rather than a binary variable; as sedation depth increases, so do monitoring requirements and the need for greater perioperative preparedness, which may partly offset the logistical advantages of local anesthesia alone.^8,11,16^ The authors’ center protocol, based on a premixed lidocaine-ropivacaine-epinephrine solution, an alternating latency sequence, and preservation of verbal responsiveness, fits coherently within this minimal-sedation paradigm and reflects the broader office-based principle that the anesthetic pathway should remain simple, reproducible, and compatible with maintenance of consciousness whenever possible.

With regard to complications, hematoma remains the central endpoint and the main parameter through which protocol safety is judged.^3,4,11^ The literature suggests that the marked variability in reported rates should be interpreted cautiously, because it is influenced by differences in outcome definitions, distinction between minor and major hematoma, need for evacuation, extent of neck dissection, and depth of sedation.^4,11,15,16^ Nevertheless, a coherent pattern emerges: blood pressure instability, anxiety, pain, and postoperative nausea/vomiting appear closely interconnected and together shape bleeding risk more than any isolated technical maneuver.^3,4^ Focused procedures performed with limited dissection and tumescent techniques appear to support low morbidity and rapid recovery in selected patients, whereas more extensive awake procedures can still maintain acceptable morbidity when the perioperative pathway is standardized and complications are promptly recognized and managed conservatively.^12-15^

In this context, the authors’ single-center finding of an overall complication rate of 5.13%, all within the deep plane subgroup, should be interpreted with caution because of the limited detail on individual adverse events available in the summary, but it remains consistent with the broader literature suggesting that a structured local-anesthesia pathway with pharmacologic preparation and monitoring may translate into a limited adverse-event profile. A recurring theme is that bleeding risk is strongly influenced by modifiable perioperative factors, particularly blood pressure instability, anxiety, pain, coughing, nausea, and vomiting.^3,4^ A local-anesthesia pathway could theoretically reduce some triggers, such as emergence hypertension and postoperative nausea, but only if intraoperative anxiety and discomfort are proactively managed.

The use of etamsylate in the present pathway reflects an institutional preference and should be interpreted as part of a multimodal perioperative strategy rather than as evidence of superiority over other hemostatic agents. Etamsylate is used in some clinical settings to support capillary hemostasis and reduce microvascular bleeding, with proposed effects on endothelial stability and platelet-related hemostasis.^17^

Patient-reported outcome measures, or PROMs, remain an important gap. Across the included studies, PROM collection is absent or nonstandardized, and PROMs were not systematically captured in our retrospective dataset. Accordingly, the present work primarily informs feasibility and safety signals, while the patient-experience dimension of awake surgery remains insufficiently characterized. Notably, all procedures in our institutional series were primary facelifts, excluding revision surgery as a potential contributor to bleeding risk within our dataset.

This study has limitations. Its retrospective design limits the granularity of anesthesiologic and hemodynamic data, pain scores, patient-reported outcomes, and minor adverse events. The procedures were not limited to a single facelift technique, which precludes technique-specific safety comparisons.

## CONCLUSIONS

Facelift under local anesthesia is feasible within a structured pathway centered on appropriate patient selection, standardized infiltration, vigilant hemodynamic monitoring, and proactive management of pain and nausea/vomiting. Local anesthesia with minimal sedation may be preferred when the surgical burden is predictable and patient cooperation is reliable. General anesthesia is more appropriate when cooperation is doubtful, airway control is a priority, hemodynamic instability risk is high, or procedural complexity/duration is expected to be prolonged or unpredictable. Future work should standardize operative time reporting, hematoma severity grading and include validated PROMs to guide transferable decision-making across settings.
